# From loneliness to connection: the formation mechanism of loneliness, job search stress, and organizational belonging among students approaching graduation in a context of employment contraction

**DOI:** 10.3389/fpsyg.2026.1754280

**Published:** 2026-03-27

**Authors:** Lu Han, Xuanle Si, Haoyu Wang, Xinchen Leng

**Affiliations:** 1 School of Humanities, Tarim University, Xinjiang, China; 2 School of Humanities and Arts, Xinjiang University of Political Science and Law, Xinjiang, China; 3 Liuzhou Polytechnic University, Liuzhou, Guangxi, China; 4 College of Social Sciences and Humanities, Northeastern University, Seattle, WA, United States

**Keywords:** anticipated organizational belonging, job search stress, loneliness, mediation, moderation, social support

## Abstract

This study examines how university students respond psychologically to an increasingly uncertain employment environment by exploring the relationships among loneliness, job search stress, anticipated organizational belonging, and perceived social support during the transition from university to work. Anticipated organizational belonging is understood here as expectations of inclusion in a future organization rather than feelings of attachment to an existing group. Drawing on belongingness theory and stress appraisal perspectives, survey data from 390 final year students in China were analyzed using structural equation modeling. The results show that loneliness was positively related to job search stress, and job-search stress accounted for part of the association between loneliness and anticipated organizational belonging. This pattern suggests that experiences of social disconnection may coincide with heightened sensitivity to employment uncertainty and a stronger orientation toward future organizational attachment. Job search stress was also positively associated with anticipated organizational belonging, indicating that under competitive and uncertain entry conditions, stress may be accompanied by greater attention to organizational stability and institutional placement. Perceived social support was positively related to anticipated organizational belonging and weakly negatively related to job-search stress, but it did not moderate the relationship between stress and belonging. Overall, the findings do not support a simple deficit interpretation in which loneliness and stress necessarily weaken belonging. Instead, anticipated belonging during the school-to-work transition appears to function as a forward looking evaluation that can increase alongside both stress and loneliness. Because the data are cross-sectional and self-reported, the results should be interpreted as associations rather than causal effects.

## 1 Introduction

### 1.1 Changes in employment patterns and the intensification of psychological loneliness among youth

Over the past several years, labor-market fluctuations have fundamentally reshaped the conditions under which young people prepare to transition from university into full-time employment. Periods characterized by unstable youth employment prospects and limited entry opportunities have not only altered career trajectories but have also weakened young people’s social embeddedness. Recent cross-national evidence shows that employment insecurity among young adults is associated with reduced social integration, lower well-being, and heightened psychological strain, indicating that labor-market uncertainty operates as a broader social and psychosocial context rather than merely an economic condition (Yang et al., 2024). Research on unemployment and social isolation helps clarify how these dynamics unfold. Morrish and Medina-Lara (2021), using propensity-score matching, showed that unemployment is associated with higher levels of social isolation and emotional loneliness and may actively reduce job seekers’ engagement in social activities and job-search behaviors, thereby reinforcing isolation within competitive labor markets. Evidence from longer-term and cross-national investigations points to similar processes. Kim (2024) found that prolonged unemployment strengthened perceptions of social exclusion and undermined self-worth, contributing to lower life satisfaction and more persistent loneliness, and Friehe and Pfeifer (2025) reported that unstable or irregular employment was associated with sustained reductions in well-being and heightened feelings of solitude across national contexts. Taken together, this body of research positions employment as a central mechanism of social integration while simultaneously revealing that employment instability is closely intertwined with diminished relational resources and psychological strain. However, most existing studies focus on individuals who have already entered or failed to enter the labor market, leaving comparatively less attention devoted to the anticipatory phase preceding organizational entry, when students remain embedded in academic settings yet increasingly oriented toward uncertain employment futures. Emerging evidence suggests that relational vulnerability may arise even before formal organizational membership is established. Wright and Silard (2022) observed that young employees entering highly competitive and digitally mediated workplaces often encounter weak interpersonal ties and limited team cohesion, implying that contemporary work environments may not readily satisfy belongingness needs. Among students approaching graduation, job-search pressure appears to further intensify these vulnerabilities. Mao and Zhao (2023) found that job-search stress during periods of limited job availability was associated with emotional withdrawal and social avoidance, with greater reliance on online communication amplifying loneliness, while Kwak et al. (2025) showed that job-search anxiety reduced psychological vitality and increased reliance on external social support during this transitional stage. Collectively, these findings point to a pre-entry psychological context in which restricted employment opportunities, diminishing relational resources, and rising job-search pressure converge. Yet an important theoretical tension remains unresolved: although employment is traditionally conceptualized as a primary source of social integration and organizational belonging, the very process of pursuing employment under conditions of scarcity appears to heighten loneliness, relational insecurity, and stress even before organizational entry occurs. Whether and how these pre-entry emotional experiences shape young people’s expectations regarding future organizational attachment therefore remains insufficiently understood, motivating the present study’s focus on how loneliness and job-search stress jointly influence anticipated organizational belonging during the transition from university to work.

### 1.2 The conceptual evolution of loneliness, stress, and belongingness theory

Theories of loneliness, stress, and belonging have evolved across sociology and psychology through shifting assumptions about how individuals experience social disconnection and integration. Early structural accounts conceptualized loneliness primarily as a macro-level consequence of social change. Seeman (1959) described it as emerging from conditions of powerlessness, normlessness, and meaning loss produced by rapid institutional transformation, positioning individuals as largely passive recipients of external disruption. Subsequent theoretical developments moved toward process-oriented explanations that emphasized individual regulation under strain. Cohen and Wills (1985) demonstrated that social support shapes emotional responses to stress, suggesting that loneliness represents one possible trajectory within stress coping processes rather than a fixed structural outcome. Hobfoll’s (1989) conservation of resources theory further advanced this view by proposing that individuals are motivated to protect valued resources—including social relationships—and that threats to or losses of these resources intensify stress while gradually strengthening experiences associated with loneliness. Compared with earlier structural perspectives, these models highlighted dynamic interactions between stress and relational resources, yet they primarily emphasized loss prevention and withdrawal dynamics, leaving less attention to how loneliness might activate efforts to restore connection. Organizational and identity-based approaches introduced a critical shift by reframing belonging as a motivational and identity-regulatory process. Ashforth and Mael (1989) showed that group identification stabilizes emotions and guides behavior, while Baumeister and Leary (1995) argued that the need to belong constitutes a fundamental human motivation, positioning loneliness not merely as an adverse byproduct of strain but as a functional signal that relational bonds require repair. This formulation created an important theoretical divergence: loneliness may either reflect resource depletion that constrains engagement or serve as a regulatory cue that redirects individuals toward environments promising identity, structure, and stable affiliation. Integrative frameworks in the early twenty-first century further bridged these perspectives. Self-determination theory (Ryan and Deci, 2000) emphasized relatedness as essential for effective goal regulation under uncertainty, attachment theory underscored reliance on established emotional regulation strategies during threat, and Mikulincer et al. (2003) demonstrated that insecure attachment patterns amplify loneliness by limiting perceived access to reassurance in stressful contexts. Hawkley and Cacioppo (2010) synthesized emotional, cognitive, and physiological evidence to show that persistent loneliness reflects self-reinforcing interactions between individuals and their social environments, functioning simultaneously as an outcome of social conditions and a mechanism shaping stress perception and relational evaluation. Existing research increasingly suggests that loneliness, stress, and belonging are not independent constructs, but operate as interconnected components of a broader psychological regulatory system. During periods of heightened uncertainty, such as the transition from university to the workplace, stress-related processes may heighten sensitivity to relational deficits. At the same time, loneliness can either constrain social engagement through perceived resource loss or motivate individuals to seek contexts that provide identity affirmation and stable social integration. This tension between withdrawal and affiliation-seeking under stress forms the theoretical foundation for the present study’s model linking loneliness, job-search stress, and anticipated organizational belonging. Moreover, a sense of belonging developed in higher education has been shown to serve as an important psychological resource for students’ future adaptation and organizational integration in the workplace. Similarly, the psychological burden experienced by final-year high school students during career decision-making reflects the interaction between expectations of future belonging environments and adaptive capacity (Mahmood et al., 2025). Taken together, these findings suggest that belonging, loneliness, and stress constitute a dynamic and interacting system during transitional periods, providing a theoretical basis for examining students’ psychological adaptation as they move from school to work.

### 1.3 Responding to theoretical gaps and constructing the stress-belonging integration model

Research on loneliness, job search stress, and belonging has expanded across multiple disciplinary traditions, yet these streams have largely developed independently, leaving their combined role during the transition from university to work insufficiently specified. Studies of loneliness consistently indicate that perceived social disconnection is associated with reduced emotional vitality and weaker responsiveness to interpersonal and task related cues (Ozcelik and Barsade, 2018) as well as diminished supportive exchanges and cooperative functioning (D’Oliveira and Persico, 2023), although this work has focused primarily on individuals already embedded within organizational contexts and therefore provides limited insight into how loneliness shapes orientations toward work prior to organizational entry. Research on job search and unemployment-related stress similarly emphasizes psychological strain under prolonged uncertainty, documenting links with emotional exhaustion, reduced confidence, and withdrawal from social and occupational activities (Wanberg, 2012), while meta-analytic evidence shows that job-search research has concentrated mainly on behavioral outcomes and intervention effectiveness rather than on how individuals interpret stress within broader meaning-making processes (Liu et al., 2014). Although studies of job-search self-efficacy have clarified motivational mechanisms involved in employment preparation (Kim et al., 2019), they offer limited explanation of how stress relates to expectations about future organizational environments or anticipated relational positioning. Work connecting loneliness to organizational attachment provides additional but fragmented insight, with reviews of workplace loneliness calling for greater attention to earlier career stages and pre-entry contexts (McCarthy et al., 2025), and social identity research demonstrating that access to multiple group memberships is associated with reduced loneliness and improved well-being (Cruwys et al., 2014), suggesting that belonging may involve orientation toward potential sources of affiliation rather than merely reflecting existing relational integration. Despite these advances, loneliness, job search stress, and anticipated organizational belonging have rarely been examined together within a unified developmental framework, leaving unclear whether loneliness during the university-to-work transition primarily constrains engagement through stress-related resource depletion or simultaneously motivates individuals to orient toward organizations as potential sources of identity and stability. The present study addresses this gap by focusing on anticipated organizational belonging, understood as expectations of inclusion and acceptance within a future organization rather than affective attachment to an existing group, allowing examination of how individuals evaluate prospective organizational inclusion before formal membership is established. During periods of heightened uncertainty, loneliness may heighten awareness of relational insufficiency while also increasing attention to future placement and stability, and from a stress appraisal perspective job-search stress reflects how individuals interpret employment-related demands rather than simply representing a negative psychological outcome. Under such conditions loneliness and stress may coexist with stronger expectations regarding future organizational attachment, suggesting that organizational belonging in transitional contexts may function as a forward-looking evaluative orientation rather than solely as an indicator of present interpersonal comfort. On this basis, the study proposes a theoretically ordered model in which loneliness is associated with students’ appraisal of job search demands, job search stress reflects the evaluative process through which this appraisal is expressed, and anticipated organizational belonging captures expectations of organizational inclusion formed during the transition from university to work, extending belongingness theory beyond established organizational membership and providing an integrated account of how isolation and stress relate to emerging expectations of connection during early career transitions.

## 2 Review

### 2.1 Global employment insecurity and the social-psychological challenges of emerging adults

The employment conditions that contemporary young adults encounter differ substantially from those experienced by earlier cohorts, as graduates increasingly enter labor markets characterized by unstable contracts, limited prospects for career progression, and slower transitions into stable employment. Benach et al. (2014), in their examination of precarious work, highlighted that irregular income, short employment spells, and reduced job security contribute to ongoing strain among workers. Longitudinal research has underscored the potential long-term consequences of these difficulties, with Thern et al. (2017) demonstrating that episodes of unemployment during youth are linked with subsequent increases in depressive and anxiety symptoms, suggesting that early experiences of labor-market uncertainty can have enduring emotional implications. Similarly, Rönnblad et al. (2019), in a review of several longitudinal studies, observed that individuals in unstable positions frequently report higher levels of emotional fatigue, psychological distress, and poorer overall health, a pattern that persists across different national contexts. Challenges are not limited to complete unemployment; forms of employment that fall short of full engagement also pose significant psychological burdens. Using two large UK samples, Mousteri et al. (2020) found that having insufficient working hours or experiencing a mismatch between job tasks and personal skills was associated with elevated levels of strain among young employees. Research conducted in the aftermath of the COVID-19 pandemic points in a similar direction, as Cook et al. (2021) reported that the proliferation of temporary and part-time roles left many young people uncertain about their responsibilities and less connected to colleagues, exacerbating feelings of instability. Comparative studies extend these findings across broader contexts, with Yang et al. (2024) identifying a consistent link between unemployment and multiple forms of psychological difficulty across diverse regions, while Vancea and Utzet (2016) noted that unemployment or unstable work frequently coincides with diminished social support, weaker senses of purpose, and a loosening of ties to community structures. Collectively, these circumstances influence how young adults navigate emotional pressures and maintain relationships during periods of employment insecurity, shaping both their day-to-day experiences and their broader sense of social belonging, and highlighting the interplay between labor-market conditions and the social-psychological adaptation of emerging adults.

### 2.2 The conceptual foundations of loneliness and loneliness from a social psychological perspective

Research on loneliness within social psychology has evolved alongside improvements in how these experiences are conceptualized and measured, reflecting a growing recognition that loneliness is a multidimensional phenomenon. Early work distinguished different forms of loneliness, with de Jong Gierveld and van Tilburg (2006) differentiating between emotional loneliness—arising from missing close attachment figures—and social loneliness, which reflects limited access to broader social networks. This distinction highlighted that feelings of disconnection can manifest in qualitatively different ways. Building on this foundation, DiTommaso et al. (2004) developed the SELSA-S scale, which assesses emotional, social, and family-related loneliness, thereby enabling researchers to examine how loneliness varies across types of relationships. Hughes et al. (2004) further demonstrated the reliability of loneliness measurement in large-scale surveys and identified indicators suitable for cross-age and cross-cultural comparisons. More recent studies have expanded the conceptual scope of loneliness to include dimensions beyond reduced social interaction. Van Tilburg (2020), for instance, described “existential loneliness,” a form associated with difficulties in meaning-making, value orientation, or a sense of coherence in one’s life. The rise of digital communication has introduced additional complexity, as Nowland et al. (2017) observed that social media can foster meaningful connection but that superficial online interactions may exacerbate isolation. Supporting this view, Kross et al. (2013) found that passive engagement on social media is linked to declines in well-being through processes of social comparison, illustrating the cognitive and emotional mechanisms operating in digital contexts. The COVID-19 pandemic further emphasized the structural conditions under which loneliness emerges; Loades et al. (2020) reported that movement restrictions and reduced in-person contact increased loneliness among adolescents across multiple relational domains, showing that environmental constraints can intensify disconnection. Collectively, these findings demonstrate that loneliness is shaped not only by the quantity of social interactions but also by the quality of relationships, the sense of meaning and purpose, and broader social conditions, providing insight into why emerging adults may experience heightened psychological strain when external circumstances limit opportunities for meaningful connection.

### 2.3 Stress, coping, and meaning-making in the job search process

The period during which young adults begin searching for employment is marked by substantial psychological demands, as entering the labor market involves frequent decision-making, navigating uncertainty, and assessing one’s own skills and abilities. Caplan et al. (1989) observed that these pressures can generate feelings of strain and self-doubt, particularly when individuals perceive their personal resources as insufficient, and they highlighted the importance of effective coping strategies in reducing the emotional burden of setbacks while providing job seekers with a clearer sense of direction. Subsequent research has elaborated on the role of cognitive appraisal and self-regulation in shaping job-search experiences. Kanfer et al. (2001) demonstrated that individuals’ job-search behaviors are influenced both by external circumstances and by their interpretations of the challenges they face, with difficulties in managing emotions or setting concrete goals undermining persistence under pressure. Longitudinal evidence from Moynihan et al. (2003) further indicates that higher self-efficacy supports sustained engagement in job-search activities and better preparation for interviews, which can ultimately affect employment outcomes, while Saks et al. (2015) argued that job-search confidence encompasses not only performance expectations but also beliefs about coping with stress and understanding the broader significance of the search process. Extended periods of unsuccessful job searching can exacerbate psychological strain, with Wanberg (2012) reviewing evidence that repeated setbacks often lead to declines in mood, reduced social involvement, and uncertainty regarding career direction. In line with this, Liu et al. (2014) contended that interventions focused solely on skill or behavior enhancement are limited in effectiveness, emphasizing the need for support that addresses coping strategies, motivation, and interpretation of setbacks. Neimeyer’s (2016) work on meaning reconstruction further illustrates how young adults may re-evaluate their self-worth, aspirations, and social connections when confronted with prolonged uncertainty in the job-search process. Collectively, this body of research underscores that job-search stress affects more than immediate emotional states; as emerging adults interpret their experiences and adjust their goals, they may reconfigure their self-perceptions and relationships, with some encountering heightened isolation while others experience a renewed interest in connection and belonging as they transition into working life.

### 2.4 The moderating role of organizational belonging and social support: toward an integrative framework

Scholars examining organizational belonging in stressful settings have shown that employees’ interpretations of workplace demands often depend on whether they feel supported by their organization. Eisenberger et al. (1986) observed that when employees believe their organization values their contributions and cares about their well-being, they tend to regard work pressures as more legitimate and manageable, which enhances their trust in the organization’s intentions. Subsequent studies refined this view. Rhoades and Eisenberger (2002) reported that support becomes meaningful when it is expressed through fair treatment, recognition of effort, and opportunities for emotional recovery. These forms of support help employees sustain confidence in the organization during periods of uncertainty. Kurtessis et al. (2015), drawing on meta-analytic evidence, found that support is linked with several indicators of psychological functioning, including reduced exhaustion, lower stress, and stronger identification with the organization. Research on identity within organizations shows how support shapes behavior. Sluss et al. (2008) found that when exchanges between employees and supervisors involve respect and clear communication, employees become more willing to treat organizational goals as part of their own identity. Studies on value fit provide a related perspective. Cable and Judge (1996) showed that when individuals perceive alignment between their personal values and those of the organization, they form early impressions of belonging and show smoother adjustment during career entry. This alignment helps individuals interpret the significance of organizational norms and expectations, especially in stressful moments. Support from outside the workplace also influences how individuals respond to strain. Viswesvaran et al. (1999) found that having people who can offer guidance, reassurance, or practical help reduces the emotional burden associated with demanding work situations. Theories of procedural justice contribute further insight. Tyler and Blader (2003) demonstrated that when group members are treated with fairness and respect, they are more likely to develop stable identities within the group and participate willingly in collective activities. Taken together, this research shows that different forms of support—whether from organizations, supervisors, or broader social networks—shape how young adults interpret uncertainty during job searches or early employment. Support can lessen emotional strain, strengthen ties to others, and help individuals maintain direction when their prospects feel unsettled. These findings clarify why supportive environments play an important role in the connections between stress and belonging.

## 3 Methods

### 3.1 Research design and participants

The present study employed a cross-sectional survey design to examine the associations among loneliness, job-search stress, anticipated organizational belonging, and perceived social support among university students preparing to enter the labor market. Participants were final-year undergraduate students in China who were approaching graduation and actively considering post-graduation employment, a period widely recognized as a critical transition during which students begin forming expectations about future work environments while simultaneously engaging in job-search activities. Data were collected using a structured questionnaire administered both online and during scheduled class sessions. For online administration, students accessed the questionnaire through a secure survey link, while paper-based questionnaires were distributed and completed during class with the permission of course instructors. Prior to participation, all students received a written explanation of the study’s purpose and procedures and were informed that participation was voluntary and anonymous, and completion of the questionnaire was taken as informed consent. A total of 410 questionnaires were returned, of which 390 were retained after excluding incomplete responses. The final sample showed a slight gender imbalance, which is typical of student populations. Responses on loneliness, job-search stress, anticipated organizational belonging, and perceived social support covered a broad range of values, indicating adequate variability for subsequent analyses. Because the data are cross-sectional, the analytical models are interpreted as theoretically ordered associations rather than evidence of causal relationships; mediation analysis is therefore used to evaluate whether the observed associations are consistent with the proposed theoretical framework rather than to establish temporal causality.

### 3.2 Measures

All study variables were measured using established self-report instruments with demonstrated psychometric properties in prior research. Unless otherwise specified, all items were rated on a seven point Likert scale ranging from 1 (strongly disagree) to 7 (strongly agree), with higher scores indicating higher levels of the corresponding construct. The use of self-report measures was considered appropriate because the focal variables concern subjective perceptions and psychological expectations that are not directly observable. To reduce potential response bias, participation was anonymous and respondents were informed that there were no right or wrong answers. All scales were translated into Chinese using a translation and backtranslation procedure, and minor wording adjustments were made to ensure clarity and contextual relevance for Chinese university students while preserving the conceptual meaning of the original instruments. Because several constructs were adapted to a preemployment context, particular care was taken to maintain conceptual distinctions among variables. In particular, anticipated organizational belonging was defined as expectations regarding future organizational inclusion rather than current emotional attachment or perceived job security. Evidence regarding reliability, convergent validity, and potential common method bias is reported in Section 4.1.

#### 3.2.1 Loneliness

Loneliness was assessed using a standardized scale designed to measure general perceived social disconnection rather than workplace specific loneliness, as participants had not yet entered formal organizational settings. The scale captures subjective evaluations of insufficient or unsatisfactory interpersonal relationships rather than objective social isolation or broader psychological distress. Participants responded to items reflecting feelings of social distance and relational inadequacy in everyday life. A sample item is: “I feel isolated from others.” Higher scores indicate greater perceived loneliness.

#### 3.2.2 Job-search stress

Job search stress was measured using a validated scale capturing participants’ overall psychological strain related to job-seeking activities. Although job-search stress may conceptually involve multiple aspects, such as employment uncertainty, competitive pressure, and performance related anxiety, the present study conceptualizes stress as a general appraisal of job-search demands rather than as separate stress sources. This approach follows appraisal-based stress perspectives, which view stress as an integrated evaluation of situational demands relative to perceived coping resources. Consistent with prior research indicating that these experiences jointly contribute to an overall stress response during job search (Mao and Zhao, 2023; Kwak et al., 2025), the construct was modeled as a single latent factor representing the overall intensity of perceived job search strain. A sample item is: “Searching for a job makes me feel anxious and overwhelmed.” Higher scores reflect greater perceived stress.

#### 3.2.3 Anticipated organizational belonging

Anticipated organizational belonging was assessed using a scale designed to capture participants’ expectations of future social inclusion, acceptance, and identification within prospective work organizations. The construct reflects a forward looking orientation that develops prior to formal organizational entry and represents anticipated relational integration rather than current emotional attachment. Accordingly, anticipated organizational belonging is conceptually distinct from affective belonging experienced within an existing organization, generalized optimism, or perceptions of employment security. The measure focuses on individuals’ expectations regarding future organizational acceptance and social embeddedness as they transition from university to work. A sample item is: “I expect to feel a sense of belonging in the organization where I will work.” Higher scores indicate stronger anticipated organizational belonging.

#### 3.2.4 Perceived social support

Perceived social support was measured using a widely used scale assessing participants’ perceived availability of emotional and instrumental support from significant others. The measure captures subjective beliefs about accessible support resources rather than the objective size of one’s social network or the frequency of social interaction. Items assess the extent to which individuals believe they can rely on others when facing difficulties. A sample item is: “I can count on people close to me when I need help.” Higher scores indicate greater perceived social support.

#### 3.2.5 Emotional intelligence

Emotional intelligence was included as an individual difference variable reflecting participants’ ability to perceive, understand, regulate, and utilize emotions in self and others. The scale used is a validated self-report instrument appropriate for university students. Emotional intelligence was treated as a control variable to account for individual differences in emotional regulation capacity that may independently influence experiences of loneliness and perceived stress, thereby reducing the possibility that observed associations among the focal variables reflect general emotional adjustment tendencies rather than theoretically specified relationships. A sample item is: “I can stay calm under pressure and manage my emotions effectively.” Higher scores indicate greater emotional intelligence.

### 3.3 Measurement of key constructs

All key constructs in this study were measured using validated instruments with established psychometric properties, with minor adaptations to ensure cultural and contextual relevance for Chinese university students (Table 1). The UCLA Loneliness Scale was employed to assess general perceived social disconnection, which is particularly relevant for students in the pre-employment stage who have not yet entered formal organizational settings, capturing subjective feelings of isolation and inadequacy in interpersonal relationships. Job-search stress was measured to reflect participants’ overall psychological strain during job seeking, encompassing uncertainty, competitive pressure, and performance-related anxiety, and was modeled as a single latent construct representing the general stress appraisal associated with the job-search process. Anticipated organizational belonging captured forward-looking expectations of social inclusion, acceptance, and identification in future workplaces, allowing assessment of participants’ perceptions prior to formal organizational entry. Perceived social support was included to reflect individuals’ perceived availability of emotional and instrumental assistance from significant others as a relational resource relevant to stress experiences, while emotional intelligence was incorporated as an individual difference factor likely to influence how participants perceive, regulate, and respond to stress, as well as how loneliness may relate to organizational belonging. All scales were translated and slightly reworded to maintain clarity and contextual relevance without altering the underlying constructs, and internal consistency was strong across all instruments. Confirmatory factor analyses supported the convergent and discriminant validity of these measures, indicating that the constructs were reliably captured in the study context. Because all variables were collected using self-report instruments, additional diagnostic analyses were conducted to assess potential common method bias, the results of which are reported in Section 4.1. Overall, these procedures provide a rigorous and contextually appropriate foundation for the subsequent structural analyses examining the relationships among loneliness, job-search stress, perceived social support, emotional intelligence, and anticipated organizational belonging.

**Table 1:** Measurement scales, example items, sources, and adaptations.

Construct	Example item	Source	Adaptation
Loneliness	“I feel isolated from others.”	Russell et al. (1978), UCLA Loneliness Scale	Focused on general perceived social disconnection; wording adjusted for Chinese student context
Job-Search Stress	“Searching for a job makes me feel anxious and overwhelmed.”	Mao and Zhao (2023), Job Search Stress Scale	Modeled as a single latent construct; minor wording modifications for clarity
Anticipated Organizational Belonging	“I expect to feel a sense of belonging in the organization where I will work.”	Hagerty and Patusky (1995), Sense of Belonging Instrument	Forward-looking adaptation for future organizational entry; translated into Chinese
Perceived Social Support	“I can count on people close to me when I need help.”	Zimet et al. (1988), Multidimensional Scale of Perceived Social Support (MSPSS)	Translated and minor wording adjustments for Chinese university students
Emotional Intelligence	“I can stay calm under pressure and manage my emotions effectively.”	Schutte et al. (1998), Emotional Intelligence Scale	Adapted for self-report with Chinese student population; wording refined for clarity

### 3.4 Analysis strategy

The hypothesized relationships among loneliness, job-search stress, anticipated organizational belonging, perceived social support, and emotional intelligence were examined using structural equation modeling (SEM). SEM was employed to estimate theoretically specified associations among latent constructs while accounting for measurement error. Model fit was evaluated using multiple indices, including the comparative fit index (CFI), Tucker–Lewis index (TLI), root mean square error of approximation (RMSEA), and standardized root mean square residual (SRMR). Indirect effects were estimated using bootstrapping procedures with bias-corrected confidence intervals to assess mediation patterns. Because the data are cross-sectional, the SEM analyses are interpreted as theory-consistent association models rather than tests of causal direction. Temporal ordering among variables is theoretically specified but not empirically confirmed, and alternative directional explanations cannot be ruled out.

## 4 Empirical results

### 4.1 Sample profile and overall levels of the core variables

Table 2 reports that several core constructs are all at relatively high levels within the same sample. The mean scores are 4.643 for Loneliness, 4.424 for job-search stress, and 4.513 for anticipated organizational belonging. Read together, these values indicate that higher perceived distance and pressure are not accompanied by a lower average level of belonging during the school-to-work transition. In this context, belonging does not simply mirror current relational experience; it can also reflect a stronger desire for stability and future organizational attachment when respondents consider entering employment. When perceived job opportunities are more limited, individuals may place greater weight on being accepted by an organization and securing a stable position, which can be expressed in scale responses as comparatively high belonging scores even when daily experience still includes Loneliness and pressure. The mean level of social support is 4.069, which is lower than the other focal variables and shows greater dispersion, indicating notable differences across respondents in perceived support. Notably, Loneliness and Social Support are positively correlated (*r* = 0.213, *p* < 0.01), which may appear counterintuitive. This pattern is unlikely to reflect coding errors or reversed items; rather, it reflects the nature of the measured constructs. Social support in this study captures subjective perception and network activity rather than the objective quantity of support, and it can coexist with perceived pressure or loneliness, particularly during transitional periods when usual networks change and job-search demands increase. Emotional intelligence has a mean of 4.300, placing it in the upper-middle range, suggesting that the presence of stress and Loneliness is unlikely to be explained by individual skill limitations alone and is more consistent with uncertainty associated with the transition context. Standard deviations for all variables range from 0.891 to 1.014, and each measure covers a wide observed interval. Loneliness ranges from 1.75 to 7.00, and social support from 1.25 to 6.75, indicating substantial within-sample variability and leaving adequate dispersion for subsequent association and conditional-effect analyses. The mean age is 21.45 years, with values between 18 and 25, placing respondents in the typical period of movement from education into the labor market. Overall, the descriptive results point to the coexistence of elevated pressure and Loneliness with a relatively high stated level of anticipated belonging, and in the later models, interpretation relies on coefficient signs and interval estimates rather than descriptive means alone, with this parallel pattern not treated as evidence that the variables offset one another.

**Table 2:** Descriptive statistics for the study variables.

Variable	*N*	Mean	SD	Min	Max
Loneliness	390	4.643	0.929	1.75	7.00
Job search stress	390	4.424	1.014	1.50	7.00
Organizational belonging	390	4.513	0.891	2.00	7.00
Social support	390	4.069	0.975	1.25	6.75
Emotional intelligence	390	4.300	0.958	1.50	7.00
Age	390	21.45	2.31	18	25

### 4.2 Bivariate association patterns among the key constructs

Table 3 reports strong and consistently positive correlations among Loneliness, job-search stress, and anticipated organizational belonging. The coefficient between Loneliness and job-search stress is 0.532, between Loneliness and anticipated organizational belonging is 0.647, and between job-search stress and anticipated organizational belonging is 0.675, with all coefficients reaching statistical significance. In this sample, the three constructs increase together rather than moving in opposite directions. The belonging measure therefore does not behave as a simple inverse indicator of negative experience. Instead, higher belonging scores appear alongside higher perceived pressure and distance. One reasonable reading is that, during the transition from school to work, concern with organizational entry and position security becomes more salient. Under competitive employment conditions, respondents may place greater weight on future organizational attachment and stability. As a result, reported belonging can remain high even when stress and Loneliness are also elevated. In this sense, the belonging score reflects a forward-looking orientation toward organizational inclusion rather than only a summary of current emotional state. Social support is positively related to each of the three core variables, with correlations ranging from 0.213 to 0.259. These coefficients indicate that perceived support rises with overall social involvement instead of appearing only where stress is lower. Individuals who report more interaction and connection may also encounter more comparison and opportunity evaluation, which can coexist with stronger stress and stronger expressed belonging at the same time. Emotional intelligence is positively correlated with all variables and shows its largest coefficient with social support (0.472), indicating that higher regulatory capacity is associated with stronger perceived connectedness. Taken together, the correlation matrix shows broad positive co-variation across variables rather than a split between negative and positive poles. The later path analyses are interpreted in line with these coefficient directions. Readings that assume Loneliness or loneliness and belonging are opposites are not supported by the observed correlations and would change how the results are understood.

**Table 3:** Correlation matrix of the study variables.

Variable	1	2	3	4	5
Loneliness	1.000				
Job search stress	0.532***	1.000			
Organizational belonging	0.647***	0.675***	1.000		
Social support	0.213**	0.259**	0.259**	1.000	
Emotional intelligence	0.240**	0.201**	0.284**	0.472***	1.000

*p < 0.05; **p < 0.01; ***p < 0.001.

### 4.3 Reliability and convergent properties of the measures

Table 4 provides evidence that the five constructs—Loneliness, job-search stress, anticipated organizational belonging, social support, and emotional intelligence—are measured with adequate internal consistency and convergent validity in this sample. Each construct is assessed with four items. Cronbach’s *α* ranges from 0.791 to 0.886, composite reliability (CR) from 0.757 to 0.855, and average variance extracted (AVE) from 0.565 to 0.692. Taken together, these values indicate that the items within each scale share a common core and capture the same underlying content, reducing the likelihood that responses are dominated by item-specific ambiguity. This measurement profile also matters for interpreting the results that follow. In the subsequent correlation and path models, Loneliness and job-search stress increase together, while anticipated organizational belonging also remains at a relatively high level. Given the levels of convergent validity reported in Table 4, this co-movement is less plausibly explained as measurement noise or simple overlap between poorly defined constructs. The belonging scale is a useful example. Its AVE reaches 0.692 and *α* is 0.791, suggesting that the construct is captured with substantial shared variance and acceptable internal consistency. In this sample, the scale appears to reflect a coherent evaluation of prospective organizational inclusion. This helps account for why belonging can rise alongside pressure when uncertainty is salient, since the item content is likely read in terms of future acceptance and position security rather than only present relational comfort. Cronbach’s α for job-search stress and emotional intelligence is close to 0.88, indicating that both perceived pressure and perceived self-regulatory capacity are reported with high internal consistency during the transition period. The stress measure therefore reflects a stable perceived condition within the measured time frame rather than random fluctuation across items. The emotional intelligence scale shows a similarly consistent item pattern, supporting its use as an individual-difference measure in the later models. Social support shows a relatively high α but a somewhat lower CR, while remaining within acceptable ranges. This combination suggests that responses to support items are internally related but may draw more strongly on specific sources or situations, leaving more heterogeneity than the other constructs. Such dispersion is compatible with a graduation-to-employment transition in which support may come from peers, family, or institutional channels and may differ in form and salience across individuals. Generally, Table 4 supports the use of these measures for the mechanism analysis. Later interpretations should follow the coefficient directions and uncertainty in the estimates rather than relying on intuitive expectations about which variables “should” move in opposite directions. When small coefficient differences appear across models, the first step is to consider specification choices—such as controls and entry order—before treating the differences as substantive shifts.

**Table 4:** Reliability and convergent validity indicators of the constructs.

Construct	Items	Cronbach’s *α*	AVE	CR
Loneliness	4	0.829	0.691	0.848
Job search stress	4	0.884	0.565	0.855
Organizational belonging	4	0.791	0.692	0.834
Social support	4	0.875	0.597	0.757
Emotional intelligence	4	0.886	0.580	0.804

### 4.4 Assessment of common method variance

To examine whether the observed associations among variables were influenced by common method variance (CMV), a latent method factor was added to the measurement model, with all observed indicators specified to load on both their theoretical constructs and an additional unmeasured method factor. As shown in Table 5, the baseline five-factor measurement model demonstrated acceptable fit (CFI = 0.847, TLI = 0.987, RMSEA < 0.001, SRMR = 0.057). After inclusion of the latent method factor, model fit changed only marginally (CFI = 0.850, TLI = 1.000, RMSEA < 0.001, SRMR = 0.057), yielding a ΔCFI of 0.003. This change is substantially below the commonly recommended threshold of 0.010, indicating that introducing a common method factor does not meaningfully improve model fit. In addition, the estimated variance attributable to the method factor was negligible (<1%), and the mean absolute loading on the method factor was close to zero (<0.01). Together, these results suggest that common method bias is unlikely to account for the relationships observed among loneliness, job-search stress, anticipated organizational belonging, perceived social support, and emotional intelligence. Therefore, the positive associations among the core constructs are unlikely to reflect a general distress factor or response tendency arising from the use of self-report measures, supporting the validity of the subsequent structural analyses.

**Table 5:** Assessment of common method variance using a latent method factor.

Model	CFI	TLI	RMSEA	SRMR	ΔCFI	CMV variance (%)	Mean |CMV loading|
Baseline measurement model (5-factor CFA)	0.847	0.987	<0.001	0.057	—	—	—
Model with latent CMV factor	0.850	1.000	<0.001	0.057	0.003	<1%	<0.01

### 4.5 Joint effects of loneliness and job search stress on organizational belonging

The regression estimates in Table 6 indicate that when Loneliness and job-search stress are included in the model at the same time, both remain significant positive predictors of organizational belonging, and their coefficients are of comparable size. The coefficient for Loneliness is larger (*B* = 0.567, SE = 0.045, *t* = 12.600), while job-search stress also shows a strong effect (*B* = 0.423, SE = 0.038, *t* = 11.132). The model accounts for 52.4% of the variance in belonging scores, which means that a considerable portion of individual differences in belonging is associated with these two situational variables. The key point is that the two predictors both contribute positively after controlling for each other. Higher perceived distance and higher perceived pressure are each linked with higher reported belonging in the same model. This result indicates that Loneliness and stress are not interchangeable indicators of a single underlying condition. Each variable adds separate explanatory information once the other is held constant. In the transition from education to employment, stronger pressure and stronger perceived distance can coincide with a greater emphasis on obtaining a stable organizational position. Under these circumstances, belonging ratings appear to capture how strongly respondents value future organizational attachment and position security. The larger coefficient for Loneliness suggests a closer link with concerns about one’s place and acceptance within an organization, whereas job-search stress is more closely tied to the demands and strain of the search process itself. Taken together, the estimates show that negative situational experiences and belonging evaluations do not move in opposite directions in this sample and can rise at the same time. Here, belonging reflects an orientation toward stability and placement rather than only a measure of current emotional comfort. These findings provide the empirical basis for the subsequent path analysis, where belonging is treated as a context-related evaluation instead of a simple positive–negative feeling indicator.

**Table 6:** Regression estimates predicting organizational belonging.

Variable	*B*	SE	*t*	Sig.
Constant	1.234	0.156	7.923	***
Loneliness	0.567	0.045	12.600	***
Job search stress	0.423	0.038	11.132	***

*p < 0.05; **p < 0.01; ***p < 0.001.

### 4.6 The transmission role of job search stress in the pathway

**Figure 1:**
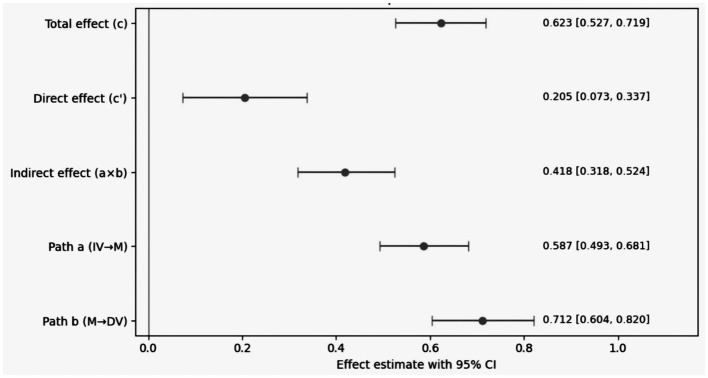
Decomposition of direct and indirect effects in the mediation model (forest plot with 95% confidence intervals).

Table 7 and Figure 1 report a mediation model in which the association between Loneliness and organizational belonging is largely carried through job-search stress rather than remaining primarily direct. The total effect is 0.623, with a 95% confidence interval of [0.527, 0.719], indicating a substantial positive association. When job-search stress is included, the direct effect drops to 0.205 [0.073, 0.337], while the indirect effect is 0.418 [0.318, 0.524], making the indirect pathway the main route of the total effect. The graphical summary is consistent with the tabled estimates. All point estimates are positive, and none of the confidence intervals include zero, indicating that the estimated effects remain positive within the sampling uncertainty reflected by the intervals. The component paths show an effect of 0.587 from Loneliness to job-search stress and an effect of 0.712 from job-search stress to belonging, with the latter larger in magnitude. This pattern indicates that belonging ratings change strongly with perceived pressure. The remaining direct effect (0.205) suggests that Loneliness also relates to belonging independently of job-search stress, which may explain the small differences observed between the main SEM and mediation-specific models. In substantive terms, the results suggest that job-search stress serves as the main route through which Loneliness is linked with higher belonging. In the school-to-work transition, Loneliness is often accompanied by stronger concerns about placement and opportunity security, and these concerns are reflected in stress reports. Belonging, in turn, captures a stronger emphasis on future organizational acceptance and stability, which allows higher pressure and higher belonging to appear together in the same period. Overall, the numerical agreement between the table and the figure supports treating this as a layered transmission pattern and motivates the later analyses that examine how the strength of these effects varies across contextual conditions. Note that coefficients reported in Table 5 (simultaneous multiple regression) are not directly comparable to c′ in Table 6 (bootstrapped mediation decomposition), because the mediation model estimates the direct effect conditional on the mediator within a decomposition framework and uses bootstrapped standard errors/intervals. Therefore, differences in point estimates reflect differences in estimation targets rather than model inconsistency.

**Table 7:** Decomposition of direct and indirect effects.

Path	Effect	SE	95% CI	Sig.
Total Effect (c)	0.623	0.049	[0.527, 0.719]	***
Direct Effect (c’)	0.205	0.067	[0.073, 0.337]	**
Indirect Effect (a × b)	0.418	0.052	[0.318, 0.524]	***
Path a (IV → M)	0.587	0.048	[0.493, 0.681]	***
Path b (M → DV)	0.712	0.055	[0.604, 0.820]	***

*p < 0.05; **p < 0.01; ***p < 0.001.

### 4.7 Relationship shifts under social support conditions

**Figure 2:**
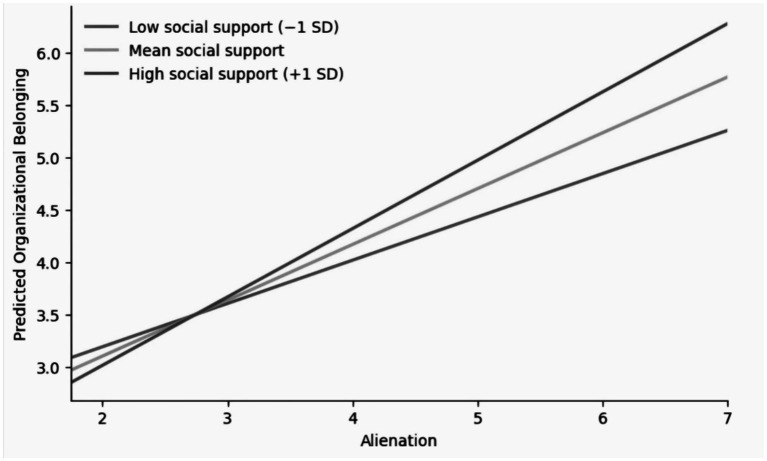
Simple slopes of the association between loneliness and organizational belonging at different levels of social support.

Table 8 reports an interaction model in which the positive association between Loneliness and organizational belonging remains after social support is added and becomes stronger at higher levels of support. The main effect of Loneliness is 0.534 (SE = 0.047, *p* < 0.001). Social support is also positively related to belonging (0.234, SE = 0.051, *p* < 0.001). The interaction coefficient is 0.123 (SE = 0.053, *p* < 0.05). Together, these estimates show that social support does not reduce the link between Loneliness and belonging. At higher support levels, the slope relating Loneliness to belonging is steeper. The simple-slope plot in Figure 2 shows this difference directly. The regression line for the high-support group has a larger slope, while the line for the low-support group is flatter. A given increase in Loneliness corresponds to a larger change in belonging scores when support is high than when support is low. Social support is often described as a buffering factor, but in these estimates it is associated with a stronger connection between Loneliness and belonging rather than a weaker one. When support networks are more available and interaction is more frequent, respondents appear more likely to evaluate their situation with reference to future organizational placement and stability. Under these conditions, higher Loneliness is accompanied by higher reported belonging. Where support is limited, Loneliness is more weakly related to belonging scores. Reports of Loneliness in lower-support settings show a smaller corresponding shift in belonging. In this sample, social support is linked to how strongly Loneliness and belonging move together, not only to lower emotional strain. The results indicate that belonging during the transition period is closely tied to social context and available support resources, and it should not be treated as simply the opposite pole of negative feeling.

**Table 8:** Interaction model with social support as moderator.

Variable	*B*	SE	*t*	Sig.
Loneliness	0.534	0.047	11.362	***
Social support	0.234	0.051	4.588	***
Interaction (IV × MOD1)	0.123	0.053	2.321	*

*p < 0.05; **p < 0.01; ***p < 0.001.

### 4.8 Path consistency under standardized specifications

Table 9 reports standardized regression coefficients for Loneliness and job-search stress predicting organizational belonging, which allows their effects to be compared on the same scale. Loneliness shows a standardized coefficient of 0.445 (SE = 0.041, *t* = 10.854, *p* < 0.001), and job-search stress shows a standardized coefficient of 0.512 (SE = 0.039, *t* = 13.128, *p* < 0.001). Both remain statistically significant in the same model, indicating that the positive associations do not depend on the original measurement units. The standardized estimate for job-search stress is slightly larger than the estimate for Loneliness. With both predictors expressed in standard deviation units, belonging is more strongly associated with the stress measure. This ordering is consistent with the mediation results, where the indirect effect through job-search stress represents a larger portion of the total effect. At the same time, Loneliness remains close to job-search stress in magnitude. This indicates that Loneliness retains a distinct association with belonging even when stress is included. Belonging is therefore related to both perceived distance and perceived pressure within the same model. Taken together, the standardized coefficients show that higher belonging scores can coincide with higher Loneliness and higher stress in this sample. This is not consistent with reading belonging as a simple indicator of immediate emotional comfort. In the school-to-work transition, belonging ratings appear to reflect evaluations of prospective organizational inclusion and stability, which can rise alongside pressure and perceived distance. These standardized results also inform the later discussion. They point to the need to explain why belonging is more strongly tied to stress than to Loneliness and to clarify how pressure is linked with higher reported belonging during the transition period, rather than assuming that negative experiences necessarily correspond to lower belonging.

**Table 9:** Standardized regression coefficients.

Variable	*β*	SE	*t*	Sig.
Loneliness (std)	0.445	0.041	10.854	***
Job search stress (std)	0.512	0.039	13.128	***

*p < 0.05; **p < 0.01; ***p < 0.001.

### 4.9 Model stability and collinearity diagnostics

The collinearity statistics in Table 10 show that the predictors in the model remain sufficiently distinct from one another. Variance inflation factors for Loneliness, job-search stress, organizational belonging, social support, and emotional intelligence range from 1.123 to 1.456, and tolerance values range from 0.687 to 0.891. These values fall within commonly accepted reference ranges and indicate that multicollinearity is not a concern in the current model. The estimated regression coefficients are therefore unlikely to be unstable or to display sign reversals due to predictor overlap. This result is relevant for interpreting the earlier findings. The positive correlations among Loneliness, stress, and belonging are not simply a consequence of the variables measuring the same content. The predictors are related, but each retains its own contribution when entered into the model. This point is important because social support and emotional intelligence are often treated as resource-related variables in contrast to Loneliness and stress. If these measures overlapped too strongly, their coefficients would be difficult to distinguish in regression analysis. The observed VIF and tolerance values indicate that this level of overlap is not present here. Because the predictors remain separable in the model, differences in coefficient size can be read as differences in association rather than as statistical artifacts of collinearity. The variable set in this study reflects several related but non-redundant dimensions, including situational strain, perceived distance, support resources, and regulatory capacity. Under these conditions, the joint increase of belonging with Loneliness and stress represents a substantive empirical pattern, not a byproduct of measurement overlap.

**Table 10:** Multicollinearity and diagnostic statistics.

Variable	VIF	Tolerance	Assessment
Loneliness	1.234	0.810	Acceptable
Job search stress	1.456	0.687	Acceptable
Organizational belonging	1.123	0.891	Acceptable
Social support	1.345	0.744	Acceptable
Emotional intelligence	1.267	0.789	Acceptable

## 5 Discussion

### 5.1 Counterintuitive positive association between loneliness and organizational belonging

The positive association between loneliness and anticipated organizational belonging observed in this study may initially appear counterintuitive, particularly in light of classical belongingness theory, which generally predicts that unmet affiliation needs reduce perceived social connection. However, this finding becomes more interpretable when considered within the school-to-work transition context and in relation to the conceptual distinction between experienced belonging and anticipated organizational belonging. The construct examined here refers to expectations regarding future inclusion in a prospective organization rather than affective attachment to an existing social group; consequently, higher belonging scores do not necessarily indicate current relational satisfaction but may instead reflect heightened attention to future organizational placement and social integration under uncertainty. Prior research suggests that experiences of social distance or labor-market instability can simultaneously increase perceived isolation while strengthening individuals’ motivational focus on securing stable institutional affiliation. Studies by Morrish and Medina-Lara (2021), Friehe and Pfeifer (2025), and Kim (2024), for example, show that extended job-search periods and unemployment are associated not only with elevated loneliness but also with stronger concern for organizational attachment and position security. In this sense, loneliness during transitional periods may coexist with stronger orientation toward anticipated inclusion rather than reduced belonging per se. Rather than contradicting belongingness theory, this pattern may extend it to a pre-entry context: loneliness can function as a regulatory signal indicating unmet affiliation needs (Baumeister and Leary, 1995), which may redirect attention toward environments expected to provide future acceptance and stability. Consistent with Hawkley and Cacioppo’s (2010) account of loneliness as reshaping social cognition and evaluation processes, individuals experiencing relational distance may evaluate future organizational contexts as potential sources of identity anchoring and social integration. Alternative explanations must also be considered. If anticipated belonging merely reflected generalized optimism or a positive response tendency, similar associations would be expected across all constructs; however, differentiated relationships among variables, together with evidence from measurement validity and common method variance diagnostics, suggest that anticipated organizational belonging represents a distinct evaluative orientation rather than a general affective bias. The mediation results further indicate that job-search stress plays an important role in this association, consistent with research showing that sustained engagement in job-search activities and repeated social comparison shape motivational focus and future-oriented evaluation (Mao and Zhao, 2023; Kwak et al., 2025). Under conditions of heightened employment uncertainty, organizational belonging may therefore function less as an indicator of present interpersonal comfort and more as an anticipatory assessment of future placement and institutional integration. Accordingly, the positive association identified in this study should be interpreted as context-dependent rather than anomalous, suggesting that during the transition from university to work, belonging can operate as a forward-looking evaluative construct that increases alongside loneliness when individuals orient themselves toward organizational environments expected to provide stability, legitimacy, and social anchoring.

### 5.2 Reassessing organizational belonging under concurrent loneliness and pressure conditions

The present findings suggest that anticipated organizational belonging during the school-to-work transition cannot be interpreted as a simple indicator of interpersonal comfort or immediate social connectedness. Across both correlational and regression analyses, loneliness and job-search stress were positively associated with belonging, and each retained an independent effect when entered simultaneously into the model, indicating that perceived social distance and perceived pressure contribute distinct explanatory information rather than reflecting a single underlying emotional state. This pattern implies that belonging judgments in transitional contexts may capture evaluative orientations toward future organizational placement rather than solely reflecting current relational experience. Under conditions of labor-market uncertainty, individuals approaching organizational entry may place increasing emphasis on securing a stable institutional position that provides legitimacy, acceptance, and role clarity. In this setting, the organization functions not only as a social environment but also as a symbolic anchor representing anticipated stability and integration. Consequently, loneliness and stress may coexist with stronger expressed belonging because belonging reflects expectations about future inclusion and positional security in addition to interpersonal warmth. Job-search stress is consistent with this interpretation, as it reflects sustained engagement in evaluative activities, repeated comparison with peers, and ongoing attention to qualification and placement outcomes, all of which orient individuals toward future organizational membership. Rather than indicating emotional contradiction, the observed pattern suggests a contextual shift in the meaning of belonging during transitional periods: belonging encompasses both experiential affiliation (feeling accepted in present relationships) and anticipatory affiliation (valuing future organizational integration). In stable organizational environments these dimensions typically converge, but during high-uncertainty transitions the anticipatory dimension may become more salient, allowing loneliness and pressure to increase alongside belonging without conceptual inconsistency. This reconceptualization contributes to belongingness theory by extending it beyond established group membership and demonstrating that belonging can operate as a forward-looking evaluative construct shaped by uncertainty and institutional expectations. Accordingly, the results indicate that anticipated organizational belonging reflects not merely emotional comfort but also orientation toward institutional anchoring, helping explain why higher loneliness and stress may coincide with stronger belonging evaluations and suggesting implications for later engagement, commitment formation, and adaptive coping during early career transitions.

### 5.3 The central role of job search stress in the formation pathway of belonging judgments

The mediation analyses indicate that the association between loneliness and anticipated organizational belonging is primarily organized through job-search stress, with the indirect component accounting for a larger proportion of the overall association than the remaining direct link. Rather than implying a temporal or causal sequence, this pattern should be interpreted as a theory-consistent decomposition of associations within the proposed conceptual ordering. In the present sample, perceived social distance is more strongly connected with belonging evaluations when it co-occurs with heightened job-search pressure, suggesting that respondents’ expectations of organizational inclusion are closely aligned with how they appraise employment-related demands and competitive conditions. Higher loneliness is associated with higher reported stress, and belonging judgments are in turn more strongly related to stress levels, indicating that evaluations of future organizational attachment are embedded in ongoing interpretations of search-related challenges rather than reflecting interpersonal distance alone. During the transition from university to work, job-search stress captures sustained engagement in application activities, repeated social comparison, and continuous evaluation of qualifications and prospects, all of which direct attention toward future placement and institutional positioning. Under these conditions, belonging appears less as a pure indicator of relational comfort and more as an evaluative orientation shaped by perceived demands and anticipated role acquisition. Importantly, the persistence of a statistically significant direct association between loneliness and belonging indicates that stress does not fully account for their relationship; instead, loneliness and stress represent partially independent yet complementary dimensions linked to belonging judgments. Job-search stress may therefore be understood as a proximal experiential context through which broader feelings of social distance become associated with expectations of organizational inclusion, while loneliness reflects a more general perception of relational insufficiency. Interpreted in this way, the mediation results do not establish a developmental mechanism but instead clarify how multiple psychological experiences jointly structure belonging evaluations during a period characterized by uncertainty, risk, and opportunity in the school-to-work transition.

### 5.4 Social support as a contextual amplifier of the loneliness–belonging link

The moderation results diverge from the traditional stress-buffering assumption, which predicts that social support should weaken the impact of adverse experiences. In the present study, however, higher levels of perceived social support strengthened rather than attenuated the positive association between loneliness and anticipated organizational belonging. This pattern does not necessarily contradict the buffering model but instead suggests boundary conditions regarding the functional role of support during transitional contexts. Classical stress-buffering perspectives primarily describe situations in which support reduces emotional strain by providing reassurance or coping resources. In contrast, during the transition from university to work, social support may operate not only as an emotional resource but also as an informational and interpretive context that shapes how individuals evaluate their future positioning. When support networks are more active and accessible, individuals are exposed to greater discussion of career plans, peer comparisons, feedback about opportunities, and expectations regarding organizational entry. Under these conditions, loneliness may be interpreted less as a purely negative emotional state and more as an indicator of not yet having secured a recognized organizational role. As a result, evaluations of anticipated organizational belonging become more strongly aligned with experiences of uncertainty and comparison, producing a steeper association between loneliness and belonging at higher support levels. Rather than canceling adverse experience, support appears to increase the salience of placement-related concerns by embedding individuals within richer informational and social-evaluative environments. Conversely, when perceived support is limited, loneliness remains more closely tied to personal emotional discomfort and less connected to future-oriented organizational evaluation, resulting in a weaker statistical association with belonging. Interpreted in this way, social support functions as a contextual amplifier that strengthens the linkage between experience and evaluative judgment rather than as a universal emotional buffer. This interpretation is consistent with appraisal-based perspectives suggesting that social environments influence how individuals construe uncertainty, and it helps explain why belonging in this study behaves more as a forward-looking assessment of organizational placement than as a simple indicator of present relational well-being.

## 6 Conclusion

Taken together, the findings invite a reconsideration of how organizational belonging is conceptualized during periods of transition and uncertainty. Rather than functioning solely as an outcome of fulfilled affiliation needs, anticipated organizational belonging may also emerge as an evaluative orientation through which individuals interpret risk, uncertainty, and future placement within structured social systems. In the school-to-work transition, belonging appears to operate not only as an emotional state grounded in present interpersonal experience but also as a forward-looking regulatory assessment linked to identity stabilization and anticipated institutional anchoring. This perspective helps reconcile the seemingly paradoxical coexistence of loneliness, stress, and stronger belonging expectations observed in the present study, suggesting that under conditions of uncertainty, individuals may orient more strongly toward organizational inclusion precisely when relational insecurity becomes salient. By situating belonging within a dynamic transition context, the study extends belongingness theory beyond established group membership and highlights how affiliative motivations may shift from experiential comfort toward prospective positioning. Although the cross-sectional design limits causal interpretation, the results point toward a broader research agenda in which belonging is examined as a context-sensitive evaluative process that evolves across pre-entry and post-entry stages of organizational life. Future longitudinal and multi-source investigations may further clarify how experiential belonging and position-oriented belonging diverge, converge, or transform over time, thereby advancing a more temporally grounded understanding of organizational attachment formation. In this sense, the present study contributes not only empirical evidence but also a conceptual reframing: belonging under uncertainty may be less a reflection of present connection than an adaptive orientation toward anticipated inclusion in emerging social and institutional futures.

## Data Availability

The raw data supporting the conclusions of this article will be made available by the authors, without undue reservation.
